# 588 Early Physical Therapy Improves Burn Rehabilitation: Nationwide Study of Patients ≤30% Total Burn Surface Area

**DOI:** 10.1093/jbcr/irae036.222

**Published:** 2024-04-17

**Authors:** Christopher G Richter, Sunskruthi Krishna, Suhaib Shah, Carolina Segura, Yash Ramgopal, Jean Pierre Durand, Georgiy Golovko, Oscar E Suman-Vejas, Steven E Wolf, Amina E I Ayadi, Juquan Song

**Affiliations:** University of Texas Medical Branch, Galveston, TX; University of Texas Medical Branch at Galveston, Galveston, TX; University of Texas Medical Branch, Blocker Burn Unit, Galveston, TX; University of Texas Medical Branch, Galveston, TX; University of Texas Medical Branch at Galveston, Galveston, TX; University of Texas Medical Branch, Blocker Burn Unit, Galveston, TX; University of Texas Medical Branch, Galveston, TX; University of Texas Medical Branch at Galveston, Galveston, TX; University of Texas Medical Branch, Blocker Burn Unit, Galveston, TX; University of Texas Medical Branch, Galveston, TX; University of Texas Medical Branch at Galveston, Galveston, TX; University of Texas Medical Branch, Blocker Burn Unit, Galveston, TX; University of Texas Medical Branch, Galveston, TX; University of Texas Medical Branch at Galveston, Galveston, TX; University of Texas Medical Branch, Blocker Burn Unit, Galveston, TX; University of Texas Medical Branch, Galveston, TX; University of Texas Medical Branch at Galveston, Galveston, TX; University of Texas Medical Branch, Blocker Burn Unit, Galveston, TX; University of Texas Medical Branch, Galveston, TX; University of Texas Medical Branch at Galveston, Galveston, TX; University of Texas Medical Branch, Blocker Burn Unit, Galveston, TX; University of Texas Medical Branch, Galveston, TX; University of Texas Medical Branch at Galveston, Galveston, TX; University of Texas Medical Branch, Blocker Burn Unit, Galveston, TX; University of Texas Medical Branch, Galveston, TX; University of Texas Medical Branch at Galveston, Galveston, TX; University of Texas Medical Branch, Blocker Burn Unit, Galveston, TX; University of Texas Medical Branch, Galveston, TX; University of Texas Medical Branch at Galveston, Galveston, TX; University of Texas Medical Branch, Blocker Burn Unit, Galveston, TX; University of Texas Medical Branch, Galveston, TX; University of Texas Medical Branch at Galveston, Galveston, TX; University of Texas Medical Branch, Blocker Burn Unit, Galveston, TX

## Abstract

**Introduction:**

While the benefits of physical therapy (PT) in burn patient rehabilitation is highlighted to great extent in literature, there are no national estimates on the effects of time from admission to initial PT evaluation. This study aims to evaluate how expedited PT evaluations influence outcomes in ≤30% total burn surface area (TBSA) patients.

**Methods:**

A national database of deidentified patient records from 2015 to 2021 was analyzed. Thermal and chemical burn patients were categorized based on the time from initial injury to PT evaluation: < 48 hours (early) or ≥ 48 hours (delayed). The study included patients with well-documented injury severity of ≤30% TBSA, while those with unspecified burn injuries or respiratory tract involvement were excluded. The outcomes, measured at one year, included continued pain, disability, psychological burden, and mortality. Analysis was performed using independent z-tests with a set value of p< 0.05.

**Results:**

The study included 2,386 patients with 58.6% evaluated by PT in the early cohort and 41.4% in the delayed cohort. The mean number of PT sessions per patient was consistent between both groups (6 sessions). At one year, the early evaluation cohort demonstrated significantly lower rates of pain (RR=0.579; CI=0.437-0.677; p< 0.0001), psychological burden (RR=0.702; CI=0.547-0.902; p=0.0052), and disability (RR=0.464; CI=0.234-0.855; p=0.0122). No significant difference in mortality was observed.

**Conclusions:**

This study highlights the significant benefits of expedited PT evaluation within 48 hours of admission for patients with ≤30% TBSA. The timely initiation of PT not only reduces pain and disability but also diminishes psychological burden at one year among patients with similar injuries. These findings emphasize the importance of prompt interprofessional integration for improved quality of life outcomes in burn patients.

**Applicability of Research to Practice:**

This study offers a focused examination of the tangible benefits associated with early PT evaluation in mild to moderate burn patients. The findings have the potential to substantially impact interprofessional standards of practice and patient care protocols.

